# Homœopathy

**Published:** 1842-10

**Authors:** 


### HOMŒOPATHY.

We have been considerably entertained by some observations on this grandest of all grand humbugs, which we find in Lea & Blanchard's Medical Intelligencer copied from the *Physiologie du Medicin* of Louis Huart. The writer after telling us that the essence of the Homœopathic doctrineis contained in the aphorism,*similia similibus curantur*, gives some instances of miraculously prompt cures, and proceeds in this wise—we translate literally:

"It is not only men that homœpathic physicians treat with the success described above. They likewise bestow their cares upon quadrupeds generally—even upon asses. However, we do not think they do so always in pursuance of their famous maxim *similia similibus*—that would really involve too much of Christian and homœopathic humility."

Then follows an illustration which is inimitable of its kind: a man with an enormous quantity of cravat, and proportionally small quantity of hair, is seated, with spectacles on nose—which last feature has a most inquisitive perk—peering intently on the protruded tongue, and feeling the pulse in the sinister fore-leg of a long-eared animal that lies in graceful reclination before him, with a most dolorous and *asinine* expression of countenance.

"Another fundamental point of the doctrine in question is, not to administer internal remedies except in infinitesimal does. This has some advantage, for without it the homœopathic practice would be much more dangerous to the public."

There is a good deal of truth, mayhap, in that last observation. After a lively and graphic description of the preparation and administration of one of these small doses, our author proceeds:

"With a lozenge [*tablette*] of jujube, the homœpathic physician would form potions to cure a whole regiment of cuirassiers affected with the whooping-cough."

But the richest conceit is the following:

"I firmly believe that the homœopathic doctrine was originally invented by a physician that had suffered a refusal at the hand of an apothecary's daughter; and from the anger of this frantic lover has arisen the system which must inevitably ruin those philanthropic establishments where they sell liquorice and senna at a profit of eight hundred per cent!"

What say you to that, reader? Is it not droll, and above all, is it not exceedingly Gallic? Apothecaries' daughters should be more circumspect.

"Hence the pharmaceutists have sworn a mortal hatred to the homœopathists, who have been obliged to prepare and vend for themselves their little packets of white powder.

Nobody would find much fault with this, if unfortunately these small, infinitely small packets of white powder did not cost as much as the great bottles of drugs under the old system!

If this is complained of, the homœopathist answers: *Every body must live!* Unfortunately this phrase is not applicable to their patients!"

Can any one tell us where to procure Huart's *Physiologie du Medicin?* It must be a rare book, racy and original. The spirit of Moliere undoubtedly dwells in the author. C.

